# Editorial on Special Issue “Artificial Intelligence in Image-Based Screening, Diagnostics, and Clinical Care of Cardiopulmonary Diseases”

**DOI:** 10.3390/diagnostics12112615

**Published:** 2022-10-27

**Authors:** Sivaramakrishnan Rajaraman, Sameer Antani

**Affiliations:** Computational Health Research Branch, National Library of Medicine, 8600 Rockville Pike, Bethesda, MD 20894, USA

Cardiopulmonary diseases are a significant cause of mortality and morbidity worldwide. The COVID-19 pandemic placed significant demands on clinicians and care providers, particularly in low-resource or high-burden regions. Simultaneously, advances in artificial intelligence (AI), machine learning (ML), and the increased availability of relevant images enhanced the focus on cardiopulmonary diseases. According to the recent American Lung Association report, more than 228,000 people will be diagnosed with lung cancer in the United States alone this year, with the rate of new cases varying by state [1]. Further, heart disease is indiscriminate in ethnic and racial origin, causing mortality. Additionally, infectious diseases, such as tuberculosis (TB) often coupled with the human immunodeficiency virus (HIV) comorbidity, are found with drug-resistant strains that greatly impact treatment pathways and survival rates [2]. The screening, diagnosis, and management of such cardiopulmonary diseases have become difficult owing to the limited availability of diagnostic tools and experts, particularly in low and middle-income regions. Early screening and the accurate diagnosis and staging of cardiopulmonary diseases could play a crucial role in treatment and care and potentially aid in reducing mortality. Radiographic imaging methods such as computed tomography (CT), chest-X-rays (CXRs), and echo-ultrasound are widely used in screening and diagnosis [3,4,5,6]. Research on using image-based AI, ML, particularly convolutional neural network (CNN)-based deep learning (DL) methods, can help increase access to care, reduce variability in human performance, and improve care efficiency while serving as surrogates for expert assessment [7]. We find that significant progress has been made [5,8,9,10] in DL-based medical image modality classification, segmentation, detection, and retrieval techniques which have resulted in a positive impact on clinical and biomedical research. We wanted to capture a snapshot of these advances through a Special Issue collection of peer-reviewed high-quality primary research studies and literature reviews focusing on novel AI/ML/DL methods and their application in image-based screening, diagnosis, and clinical management of cardiopulmonary diseases. These published studies present state-of-the-art AI in cardiopulmonary medicine with an aim toward addressing this global health challenge.

Studying the articles in this collection, the reader will observe that the choice of the DL model depends largely on the characteristics of the data under study [11]. A study of the literature reveals that no individual DL model is optimal for a wide range of medical imaging modalities [12]. Despite delivering superior performance, the performance of DL models is shown to improve with the availability of meaningful data and computational resources [13]. The quality of medical images and their annotations also plays an important role in the success of DL models. The visual characteristics of medical images, viz., shape, size, color, texture, and orientation are unique compared to the natural stock photographic images [14]. The regions of interest (ROIs) concerning the disease manifestations or the organs in medical images are relatively small compared to natural images. Hence, it is crucial to select the optimal DL model for the medical image modality and problem under study. Unlike natural images, medical images and their associated labels are often scarcely available. Strategies including transfer learning [13,15] and multicenter collaboration [11] have been proposed to handle data scarcity issues. The transfer learning-based approaches are prominently used as they leverage the knowledge learned from a large collection of stock photographic images such as ImageNet [16] to improve performance and generalization in medical visual recognition tasks with a sparse collection of medical data and their associated labels. In this regard, Gozzi et al. [17] proposed the identification of the optimal transfer learning strategy for a CXR classification task. They followed a systematic procedure which is as follows: (i) Several ImageNet-pretrained CNN models were retrained on the publicly available CheXpert [18] CXR dataset. This approach facilitated learning CXR modality-specific feature representations. A study of the literature [19,20,21] reveals that the medical image modality-specific retraining of ImageNet-pretrained models demonstrates significant gains in related classification, segmentation, and detection tasks. The authors evaluated the classification performance achieved through multiple transfer learning methods such as image feature (embedding) extraction, fine-tuning, stacking, and tree-based classification using a private CXR dataset. They qualitatively evaluated performance using gradient-weighted class activation maps (Grad-CAM) [22]. In this regard, the authors demonstrated superior performance with a 0.856 area under the curve (AUC) using the image embeddings extracted from the penultimate layer of the CNN models and an averaging ensemble of the RF predictions, showcasing it as the optimal transfer learning strategy for the task under study. The Grad-CAM maps showed that the CNN models learned task-specific features to improve prediction performance.

In another study, Huang et al. [23] evaluated the gains achieved through transfer learning in a multi-label CXR classification task. They used a private CXR collection containing multiple abnormalities including aortic sclerosis/calcification, arterial curvature, consolidations, pulmonary fibrosis, enlarged hilar shadows, scoliosis, cardiomegaly, and intercostal pleural thickening, etc. The ImageNet-pretrained CNN models were retrained on the CheXpert and NIH CXR-14 [24] datasets to learn CXR modality-specific representations. The learned knowledge was transferred and finetuned for a related CXR classification task. They further evaluated the gains achieved through multiple transfer learning strategies such as the reuse of pretrained weights, layer transfer where some of the model weight layers were frozen, and model retraining, using the models trained on differently sized CheXpert and NIH CXR-14 datasets. It was observed that CXR modality-specific finetuning of the ImageNet-pretrained models, using the NIH CXR-14 dataset, demonstrated superior prediction performance with an accuracy of 0.935, compared to other models/methods. The authors recommend retraining the CNN models using multiple cross-institutional datasets for promising performance and generalization under conditions of sparse medical data and label availability.

DL models have demonstrated poor performance and generalization in cases where the distribution of the data used to train the models (source distribution) is different compared to the unseen real-world data (target distribution). This lack of generalization could be attributed to several factors including changes in image acquisition protocols, data formatting and labeling, patient heterogeneity based on age, gender, race, and ethnicity, and varying characteristics of the underlying disease manifestations, etc., between the source and target distribution [25]. The discrepancy in the characteristics of the source and target data may eventually lead to domain shift issues resulting in performance degradation and sub-optimal generalization. Under these circumstances, training and evaluating the models using the source data may not accurately reflect real-world settings. Karki et al. [26] discussed the generalization issues with the DL models that were trained to classify Drug-Resistant TB (DR-TB) manifestations from drug-sensitive TB (DS-TB) using CXRs. They observed sub-optimal classification performance with an AUC = 0.65 using an unseen test set in a CNN model that was trained on internal data. The authors observed poor localization using Grad-CAM activation maps as compared to the radiologist-annotated ROIs. Training a multi-task attention model using lesion location information from prior TB infection helped to improve classification performance (AUC = 0.68) on the blinded test set. The authors highlight differences in acquisition protocols and the variation in non-pathological and non-anatomical image attributes across the datasets that contributed to sub-optimal performance and generalization.

Mueller et al. [27] assessed the diagnostic performance of dual-energy subtraction radiography (DE) [28] in detecting pulmonary emphysema and compared it to the performance achieved using conventional radiography (CR). Pulmonary emphysema, a chronic obstructive pulmonary disease (COPD), blocks airflow in the lungs and causes breathing disorders. CT imaging is reported to be the most sensitive radiological imaging method for detecting and quantifying pulmonary emphysema [29]. The authors used the posteroanterior and lateral radiographic projections acquired from patients using CR, DE, and CT radiography imaging. Expert radiologists were involved in identifying the presence and degree of manifestations consistent with pulmonary emphysema in the DR and CR images while keeping CT as the reference standard. The specificity and recall in detecting and localizing the disease and the inter-reader consensus were measured. The authors observed a high consensus between the readers in identifying pulmonary emphysema manifestations using CR images (Kappa = 0.611) and a moderate consensus (Kappa = 0.433) using the DR images. The authors conclude that the diagnostic performance in terms of detecting, quantifying, and localizing pulmonary emphysema manifestations using CR and DE imaging was comparable.

Li et al. [30] performed a systematic review of the literature to analyze the additional effect of AI-based methods on the performance of physicians to detect cardiopulmonary pathologies using CXR and CT images. They followed the Place of Relevant Intermediary Approach (PRIMA) [31] to record different stages during their literature review process. The authors retrieved relevant literature on AI-based cardiopulmonary screening/diagnosis, published in the last 20 years, using Web of Science, SCOPUS, PubMed, and other literature archives. The authors analyzed human performance in terms of evaluation time, recall, specificity, accuracy, and AUC, in the presence or absence of AI-based assistive tools. It was observed that the average recall increased from 67.8% to 74.6% when human decisions were supplemented by AI assistive tools. A similar improvement was observed in terms of specificity (82.2% to 85.4%), accuracy (75.4% to 81.7%), and AUC (0.75 to 0.80). A significant reduction in the evaluation time was also observed with AI assistance.

In our work [32], we evaluated the gains achieved using modality-specific CNN backbones in a RetinaNet model toward detecting pneumonia-consistent manifestations with CXRs. We retrained ImageNet-pretrained DL models, viz., VGG-16, VGG-19, DenseNet-121, ResNet-50, EfficientNet-B0, and MobileNet on CheXpert and TBX11K datasets to learn CXR modality-specific features. The best-performing model architectures, viz., VGG-16 and ResNet-50, were used as the modality-specific classifier backbones in a RetinaNet-based object detection model. We used focal loss and focal Tversky loss functions to train the classifier backbones. The RetinaNet model was finetuned on the RSNA CXR [33] collection to detect pneumonia-consistent manifestations. We compared detection performance using various weight-initialization methods, viz., random, ImageNet-pretrained, and CXR modality-specific weights, for the classifier backbones. We observed that the VGG-16 and ResNet-50 classifier backbones, initialized with the CXR modality-specific weights, delivered superior performance compared to random and ImageNet-pretrained weight initializations. We further constructed a weighted averaging ensemble of the predictions of the top three performing models, viz., ResNet-50 with CXR image modality-specific weights trained with focal loss, ResNet-50 with CXR image modality-specific weights trained with focal Tversky loss, and ResNet-50 with random weights trained with focal loss, to arrive at the final predictions. We observed that weighted averaging delivered superior values for the mean average precision (mAP) metric (mAP: 0.3272), which was observed to be markedly superior to the state-of-the-art (mAP: 0.2547). We attribute this performance improvement to the key modifications in terms of CXR modality-specific weight initializations and ensemble learning that reduced prediction variance compared to the constituent models.

A study of the literature reveals that COVID-19 viral infection could cause acute respiratory distress syndrome and may lead to rapidly progressive and lethal pneumonia in infected patients [34]. The laboratory-based real-time reverse transcription polymerase chain reaction (rRT-PCR) test has been reported to be the most sensitive test for identifying COVID-19 infection [35]. However, there are several challenges reported in performing this test, some of which include high false negative rates, delayed processing, variability in test protocols, and reduced recall, among others. CT imaging has been reported to be an effective alternative in identifying COVID-19 disease-consistent evolution, manifestation, and progression [36]. AI-based methods applied to CT imaging could supplement clinical decision-making in identifying COVID-19, particularly in resource-constrained settings to facilitate swift referrals and improve patient care. Suri et al. [37] performed an inter-variability analysis by segmenting the lungs for assessing COVID-19 severity using CT images. The authors used two ground-truth (GT) annotations from different experts and trained U-Net [38] models to segment the lung regions of interest. The authors hypothesized that an AI model could be considered unbiased if the test performance reported with the models when trained on two different GT annotations lay within the 5% range. They further validated their hypothesis through empirical observations. It was observed that the difference in the correlation coefficient obtained using the models trained on two different GT annotations was below the 5% range, thereby showcasing a robust lung segmentation performance.

In another study, Wang et al. [39] measured the three-dimensional (3D) vascular diameter of the aorta and the pulmonary artery in Non-Contrast-Enhanced Chest CT Images to detect pulmonary hypertension. The authors proposed a novel two-stage, 3D-CNN segmentation pipeline to segment the aorta and pulmonary artery and measure the diameter in the 3D space. The authors reported superior segmentation performance in terms of the Dice similarity coefficient (DSC) metric in this segmentation task (0.97 DSC for the aorta and 0.93 DSC for the pulmonary artery). The authors discussed the benefits of such a segmentation approach in terms of providing a non-invasive, pre-operative evaluation of pulmonary hypertension for the optimal planning of surgery and reducing surgical risks.

Khan et al. [40] proposed a joint segmentation and classification network to detect pulmonary lung nodules in publicly available lung CT datasets. Performing unified segmentation and classification would not only help to learn and delineate the semantic regions of interest but also classify them into their respective categories. The authors used the VGG-SegNet [41] for nodule segmentation. The classification model was constructed by appending the classification layers to the VGG-SegNet encoder backbone. The extracted features from the penultimate layer of the trained model were concatenated with hand-crafted features extracted using a gray-level cooccurrence matrix (GLCM), local binary patterns (LBP), and pyramidal histogram of gradient (PHOG) algorithms. A radial basis function kernel-initialized support vector machine (RBF-SVM) classifier learned these concatenated features to improve classification performance with a 97.83% accuracy.

AlOthman et al. [42] proposed a novel feature extraction technique with minimal computational overload to detect and assess the severity of coronary artery disease (CAD) using CT images. The authors used the enhanced features from the accelerated segment test (FAST) to reduce the dimensions of the features extracted from a CNN model. The authors observed improved performance with this feature extraction method, demonstrating accuracies of 99.2% and 98.73% with two benchmark datasets. These findings highlighted the importance of optimal feature selection methods to improve model performance.

Germain et al. [43] analyzed whether CNN models could supersede the performance of experienced clinicians in diagnosing Cardiac Amyloidosis (CA) using Cine-Cardiovascular cine magnetic resonance (Cine-CMR) images. This disease results in the accumulation of amyloid fibrils in cardiac tissues that might lead to progressive cardiomyopathy. Cine imaging is a type of magnetic resonance imaging (MRI) sequence that captures motion. Cine-CMR is a sensitive diagnostic modality that is used to assess cardiac tissue characterizations and dysfunctions such as CA [44]. The preprocessed systolic and diastolic cine-CMR images were used to train a VGG-based CNN model to classify them as manifesting CA or left ventricular hypertrophy (LVH). The model performance was compared to the outputs of three experienced radiologists. The VGG-based CNN model significantly superseded (*p* < 0.05) human performance on frame-based evaluations, demonstrating an accuracy of 0.746 and AUC of 0.824 as compared to human experts (accuracy = 0.605 and AUC = 0.630). A similar performance improvement was observed in patient-based evaluations. The authors concluded that CNN models have a unique capability to identify CA manifestations in Cine-CMR images compared to trained human experts.

The electrical conductivity is observed to vary considerably among the biological tissues and the movement of gases and fluids within these tissues. Electrical impedance tomography (EIT) is a non-invasive medical imaging modality that uses surface electrodes to measure the electrical permittivity, impedance, and conductivity of biological tissues. However, there exists an inverse problem in EIT imaging in which the non-linear and noisy nature of the EIT imaging acquisition results in sub-optimal reconstruction. Recently, artificial neural networks (ANN) have gained prominence in tackling the inverse problem in EIT imaging. Rixen et al. [45] proposed an ANN model to resolve the EIT inverse problem. The authors reused the dense layers in the ANN model multiple times while considering the rotational symmetries exhibited by the EIT in the circular domain. The authors used an α-blending method to generate synthetic data and augment the training samples. Superior reconstruction performance and robustness to noise were reported with augmented training in which the ANN model demonstrated high values for the amplitude response (AR: 0.14) and low values for the position error (PE: 7.1) compared to conventional methods (AR: 0.1 and PE: 11.0).

In conclusion, the manuscripts published in this Special Issue discuss the novel, state-of-the-art methods for binary, multiclass, and multi-label classification, 2D and 3D image segmentation, object detection and localization, image reconstruction, generalization, recommendation, and inter-reader consensus analysis for identifying, segmenting, classifying, quantifying, reconstructing, and interpreting cardiopulmonary diseases using several medical imaging modalities including CT, MRI, CXRs, and EIT, among others. Nevertheless, deploying these proposed approaches in real-time settings remains an open avenue for research. We would like to express our sincere thanks to the authors for their significant contributions. We hope readers benefit from these research findings, and that the work included in this Special Issue inspires novel methods for diagnosis, treatment, and processes that could eventually promote healthcare.
